# Non-Invasive Hemodynamics Monitoring System Based on Electrocardiography via Deep Convolutional Autoencoder

**DOI:** 10.3390/s21186264

**Published:** 2021-09-18

**Authors:** Muammar Sadrawi, Yin-Tsong Lin, Chien-Hung Lin, Bhekumuzi Mathunjwa, Ho-Tsung Hsin, Shou-Zen Fan, Maysam F. Abbod, Jiann-Shing Shieh

**Affiliations:** 1 Department of Mechanical Engineering, Yuan Ze University, Taoyuan 32003, Taiwan; muammarsadrawi@yahoo.com (M.S.); mathunjwabhekie@gmail.com (B.M.); hsinht@gmail.com (H.-T.H.); 2 AI R&D Department, New Era AI Robotic Inc., Taipei 105, Taiwan; lotusytlin@neweraai.com (Y.-T.L.); lance_lin@neweraai.com (C.-H.L.); 3 Cardiovascular Intensive Care Unit, Far-Eastern Memorial Hospital, New Taipei City 220, Taiwan; 4 Department of Anesthesiology, College of Medicine, National Taiwan University, Taipei 100, Taiwan; shouzen@gmail.com; 5 Department of Electronic and Electrical Engineering, Brunel University London, Uxbridge UB8 3PH, UK; Maysam.Abbod@brunel.ac.uk

**Keywords:** non-invasive system, hemodynamics, electrocardiography, arterial blood pressure, central venous pressure, pulmonary arterial pressure, intracranial pressure, deep convolutional autoencoder

## Abstract

This study evaluates cardiovascular and cerebral hemodynamics systems by only using non-invasive electrocardiography (ECG) signals. The Massachusetts General Hospital/Marquette Foundation (MGH/MF) and Cerebral Hemodynamic Autoregulatory Information System Database (CHARIS DB) from the PhysioNet database are used for cardiovascular and cerebral hemodynamics, respectively. For cardiovascular hemodynamics, the ECG is used for generating the arterial blood pressure (ABP), central venous pressure (CVP), and pulmonary arterial pressure (PAP). Meanwhile, for cerebral hemodynamics, the ECG is utilized for the intracranial pressure (ICP) generator. A deep convolutional autoencoder system is applied for this study. The cross-validation method with Pearson’s linear correlation (R), root mean squared error (RMSE), and mean absolute error (MAE) are measured for the evaluations. Initially, the ECG is used to generate the cardiovascular waveform. For the ABP system—the systolic blood pressure (SBP) and diastolic blood pressures (DBP)—the R evaluations are 0.894 ± 0.004 and 0.881 ± 0.005, respectively. The MAE evaluations for SBP and DBP are, respectively, 6.645 ± 0.353 mmHg and 3.210 ± 0.104 mmHg. Furthermore, for the PAP system—the systolic and diastolic pressures—the R evaluations are 0.864 ± 0.003 mmHg and 0.817 ± 0.006 mmHg, respectively. The MAE evaluations for systolic and diastolic pressures are, respectively, 3.847 ± 0.136 mmHg and 2.964 ± 0.181 mmHg. Meanwhile, the mean CVP evaluations are 0.916 ± 0.001, 2.220 ± 0.039 mmHg, and 1.329 ± 0.036 mmHg, respectively, for R, RMSE, and MAE. For the mean ICP evaluation in cerebral hemodynamics, the R and MAE evaluations are 0.914 ± 0.003 and 2.404 ± 0.043 mmHg, respectively. This study, as a proof of concept, concludes that the non-invasive cardiovascular and cerebral hemodynamics systems can be potentially investigated by only using the ECG signal.

## 1. Introduction

In the intensive care unit (ICU), the most precise health monitoring system is utilized to thoroughly observe the critically ill patients. Hemodynamics is the blood physical phenomena in the circulatory system and a fundamental standard utilized in the ICU. Arterial blood pressure (ABP), pulmonary arterial pressure (PAP), and central venous pressure (CVP) are hemodynamics measures related to the cardiovascular system. Meanwhile, the intracranial pressure (ICP) is applied pressure due to the fluid inside the skull, measured for the cerebral hemodynamics system evaluation. These measures are essential for the patients in the ICU.

For the cardiovascular hemodynamics, CVP evaluation is fundamental for heart failure patients. It is significant in measuring the cardiac preload and blood volume information. However, the measurement requires the central venous catheter, which is highly invasive. Meanwhile, another important investigation of cardiovascular hemodynamics is PAP, which is the driven force given by the heart to pump the blood from the heart to the lung. Elevated PAP is detected for pulmonary hypertension. This evaluation, similar to CVP, is highly invasive through right heart catheterization. Further, ABP is the least invasive system compared to CVP and PAP in the cardiovascular hemodynamics system. For the cerebral hemodynamics, ICP is critical to the brain condition. The elevated ICP can have a significant indication for traumatic brain injury (TBI), and hemorrhagic stroke patients [1,2,3]. However, besides their benchmark accuracy, these evaluations are highly invasive procedures and potentially lead to infection [4]. Therefore, these measurements are not suitable for a monitoring system.

Recently, non-invasive technologies have been developed for the monitoring of the cardiovascular and cerebral hemodynamics systems. Specifically, for the cardiovascular-based hemodynamics, the utilization of artificial intelligence (AI) with photoplethysmography (PPG) has been widely used for ABP evaluations non-invasively. A study was applied to a single PPG signal with artificial neural networks (ANN) to predict ABP [5]. Furthermore, electrocardiography (ECG) and PPG, with ANN and long short-term memory (LSTM) methods were applied for the evaluations [6]. Backpropagation with a genetic algorithm (GA) was also used for hemodynamics evaluation [7]. Meanwhile, another study utilized ballistocardiography (BCG) and ECG alongside the PPG with convolutional neural networks (CNN) and a gated recurrent units (GRU)-based technique [8]. Additionally, other studies investigated the continuous arterial blood pressure evaluation based on the single PPG signal using the LSTM network [9] and hybrid GA-based optimization with a convolutional autoencoder [10]. However, even though the PPG provides a well-classified result of the blood pressure estimation, the sensor is very sensitive to motion artifacts for vertical movement and several typical activities [11,12].

Several previous studies have also been conducted for non-invasive CVP and PAP measurements. An ultrasound-based system was developed for the non-invasive CVP without central venous access [13]. In addition, a linear regression method by utilizing ICU patient data was implemented for evaluating the CVP signal using echocardiography with comparison to right heart catheterization [14]. Another study also performed a linear regression method from heart failure patients. The patients were under right heart catheterization for the CVP evaluation. The inferior vena cava utilizing echocardiography was applied for the input signal. For the non-invasive PAP system, a study utilized electrical impedance tomography of the input of the system [15]. This previous study was initiated for healthy subjects. However, a single-lead ECG system is more suitable compared to these methods for an intensive monitoring system.

For the cerebral hemodynamics, several previous studies were investigated. Most of them used the ABP and cerebral blood flow velocity (CBFV). A study by Jaishankar et al. [2] utilized ABP and CBFV as inputs for interpreting the ICP and the frequency-based method was selected for the evaluation. Another study applying a Bayesian-based approach was conducted by Imaduddin et al. [3] from the patients with TBI, hydrocephalus, and hemorrhagic stroke, using ABP and CBFV signals in order to evaluate the ICP. The utilization of ABP makes these previous studies less invasive, but not fully non-invasive.

In general, ECG is the more regularly used measurement to evaluate the cardiovascular activities for a longer period. Most importantly, ECG is a non-invasive system. Furthermore, it has been fundamentally applied for the physiological signal evaluation of arrhythmia [16], anesthesia [17,18], and sleep-related evaluations [19,20,21].

Prior studies have investigated the interconnection between ECG alterations and cardiovascular hemodynamics. It was an indication of increased P wave incidence from the ECG that correlates to hypertension [22]. The P wave also appeared later from the ECG, especially when the diastolic blood pressure was greater than 120 mmHg in hypertensive patients compared to the normal subjects. Another P wave phenomenon was also revealed. A study depicted that the hypertensive patients have a wider P wave compared to the normal subjects [23]. Furthermore, a higher T wave with extended PR intervals was also seen on hypertensive patients [24]. In addition, ST segment depression [25] and higher QRS amplitude also exhibited the information of hypertension [26]. Meanwhile, the ECG can be potentially used for pulmonary hypertension evaluation [27,28]. Recently, a single ECG signal has been utilized to investigate the hypertension system [29,30]. As it can be seen, some previously conducted studies have morphologically enlightened some relationship between cardiac electricity and blood pressure.

On the other hand, an ECG also shows several promising results when evaluating the cerebrovascular hemodynamics. The brain–heart interaction studies revealed several relationships between these associating signals. ECG abnormalities—increased corrected QT (QTc) interval, much higher P wave amplitude, higher QRS amplitude, and longer ST segment—appeared in patients with head injury compared to healthy subjects [31,32]. Furthermore, another study also revealed that the more obvious the abnormalities, the more deteriorated the consciousness level of the patients. Meanwhile, subarachnoid hemorrhage condition (SAH) can be seen morphologically in T and R wave abnormalities [33]. Specifically, in head trauma, abnormalities are discovered from prolonged QTc [34]. Furthermore, this previous study also depicted that the more severe SAH, the more prolonged QTc. For intracranial investigation, morphologically, some changes appeared in the ECG—U wave, T wave, ST-T segment, QT interval, J wave [35,36]. In addition, ECG is considered as the secondary effect of traumatic brain injury (TBI) [37]. 

As previously mentioned, the evaluation of hemodynamics is significant, especially for cardiovascular- and cerebral-related conditions. However, most of the precise procedures are measured invasively. Therefore, the aim of this study is to generate cardiovascular and cerebral hemodynamics monitoring systems non-invasively using the ECG signal and a deep convolutional autoencoder system.

## 2. Materials and Methods

This study used two databases from PhysioNet [38]: Massachusetts General Hospital/Marquette Foundation (MGH/MF) Waveform Database [39] and Cerebral Hemodynamic Autoregulatory Information System Database (CHARIS DB) [40]. For the hemodynamics system, the shorter window size was evaluated, and the better prediction was obtained. This compensates for the rapid change in some circumstances. However, the ECG is unpractical to be analyzed in very short period. This is due to, for normal subjects, the QRS cycle being sometimes not fully formed within a too short period. Therefore, the 2 s window of signal was selected for the input and output system. It was then combined and randomly separated from all the patients for training and testing with no overlapping selection. 

**Figure 1:**
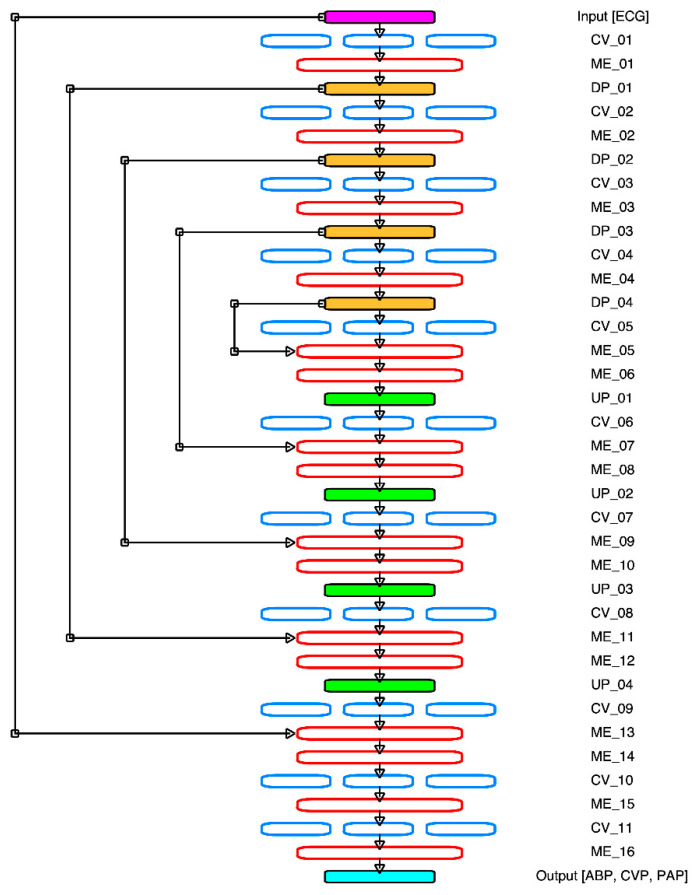
Cardiovascular hemodynamics MA-UDCAE model structure. Note: CV: convolution layer; ME: merge layer; DP: down pooling layer; UP: up sampling layer.

**Figure 2:**
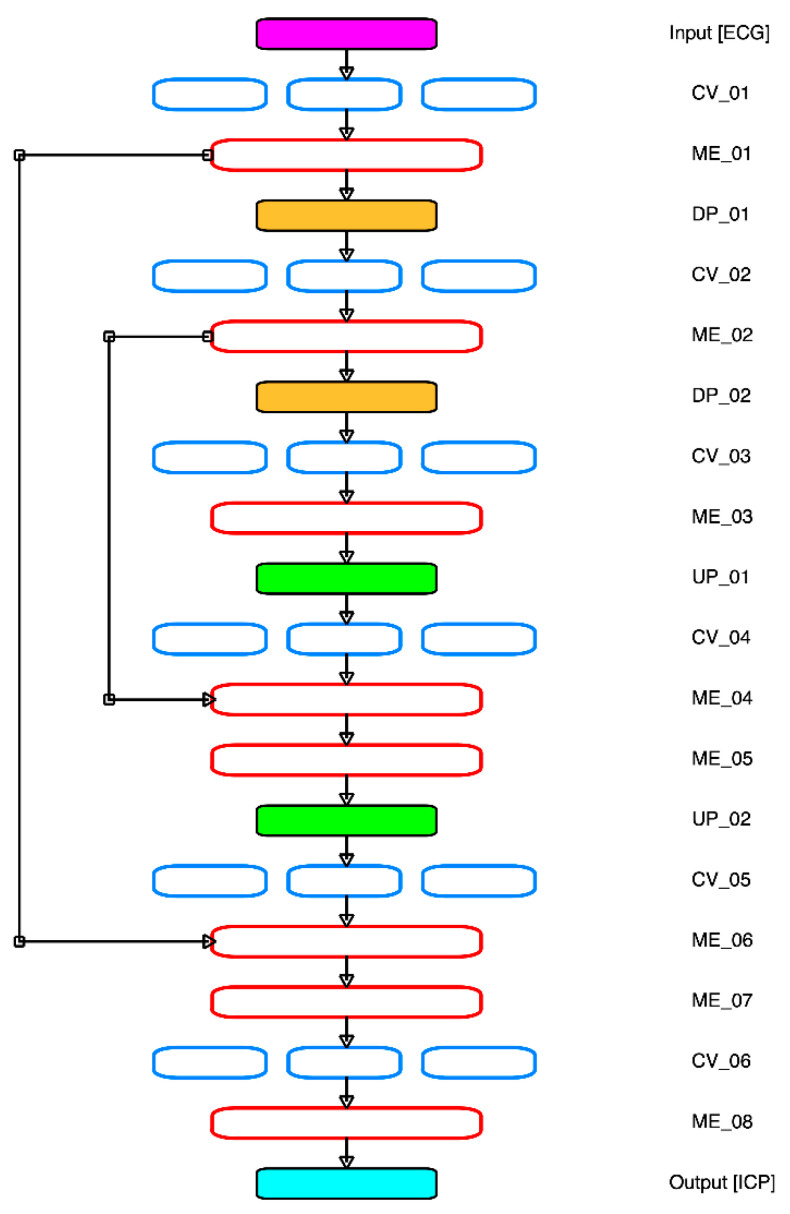
Cerebral hemodynamics MA-UDCAE model structure. Note: CV: convolution layer; ME: merge layer; DP: down pooling layer; UP: up sampling layer.

A deep convolutional autoencoder (DCAE) system was used for the signal generation model. The DCAE model is a modified model from the original U-Net model used in a biomedical image generator segmentation system [41]. This modified model deploys a multi-atrous U-Net based deep convolutional autoencoder (MA-UDCAE) system, which was originally applied by [10]. Figure 1 and Figure 2 show the MA-UDCAE model structures for both cardiovascular and cerebral hemodynamics systems. 

Figure 1 shows the convolutional autoencoder system applied to the cardiovascular hemodynamics system. For the first half, the encoder system decreases the shape of the layer. Meanwhile, the decoder increases the shape of the layer. This structure consists of several layers: convolution layer (CV), down pooling layer (DP), up sampling layer (UP), and merging layer (ME). Initially, the input of this system is the ECG. The next layer, the first convolution layer (CV_01) with multi dilation is applied. The next layer of this multi-dilation layer is merged into ME_01 followed by the down pooling (DP_01) layer. For the down pooling, this study utilizes the max pooling system. The second convolution layer (CV_02) is used and merged into the ME_02 layer. Later, the ME_02 layer is down pooled into DP_02. This block—the convolution layer, concatenation, and down pooling—is replicated until the 4th layer. For the decoder, this system is identical to the encoder system. However, the down sampling system is switched into the up sampling. Furthermore, the U-Net based system is also applied into this system. A layer in encoder is merged with the decoder layer. There are four merging layers: DP_03-ME_07, DP_02-ME_09, DP_01-ME_11, and input layer ME_14. Finally, several convolution layers are applied before the output layer, which has three channels: ABP, CVP, and PAP signals.

Figure 2 shows the cerebral hemodynamics system, which is identical to Figure 1. However, this figure has two merged layers. Furthermore, this system only has a single output channel—intracranial pressure signal (ICP). Initially for the encoder, the ECG input processed by the multi-altrous convolution system (CV_01), followed by the merging layer (ME_01), and a down pooling layer (DP_01). The next block is identical—the convolutional layer is followed by the concatenating layer and down pooling layer. For the decoder block, the first up sampling layer (UP_01) is also processed by the convolutional layer (CV_04) and concatenating layer (ME_04). Moreover, in this decoder system, the ME_02 is merged with ME_04 to form the ME_05 layer, similar to ME_07 by combining between ME_01 and ME_06 layers. Finally, the last layer is the ICP waveform.

Initially, the Massachusetts General Hospital/Marquette Foundation (MGH/MF) Waveform Database was used for the cardiovascular hemodynamics system. It contains several physiological signals: ECG, ABP, CVP, and PAP. This system was uniformly sampled for 360 Hz. From this database, this study uses only the lead II ECG signal, as the input signal, to initially generate the waveforms of ABP, CVP, and PAP, as the outputs. The utilization of the lead II ECG signal is due to this lead being mostly utilized in ECG record consideration [42]. Furthermore, the systolic and diastolic pressures of ABP and PAP were calculated by taking the maximum and minimum values, respectively, from the signal. Meanwhile, the mean CVP value was taken from averaging the 2 s CVP waveform. There were 59,401 and 14,850 of 2 sec segments, respectively, for training and testing.

Furthermore, the Cerebral Hemodynamic Autoregulatory Information System Database (CHARIS DB) was used for the intracranial pressure (ICP) evaluation. This database was sampled for 50 Hz. The input signal was the ECG signal, meanwhile the output signal was the ICP signal. A 2 s window was also selected, both for the input and output systems. Furthermore, there were 1,451,281 and 362,560 of 2 sec segments, respectively, for training and testing. Even though this database contains smaller number of patients, the data collection was much longer compared to the dataset utilized for the cardiovascular hemodynamics system.

For pre-processing the data and post-processing the result, MATLAB R2014b (MathWorks, Inc., Natick, MA, USA) was utilized. The data were initially filtered manually by visualization. The input and output signal must be appropriate for the training and testing. If either the input or output signal is noisy, both will be deleted. For training the deep convolutional autoencoder system, Python 3.6 was utilized with TensorFlow (Ver. 1.15.2) [43] and Keras (Ver. 2.3.1) under Google Colaboratory (Google Inc., 1600 Amphitheatre Parkway Mountain View, CA, USA). In more detail, the training of both cardiovascular and cerebral hemodynamics systems were conducted with the checkpoint system.

For the evaluation, a 5-fold cross-validation was performed with shuffled training data. Generally, this strategy is performed to evaluate the regularity of the data to the model. Furthermore, Pearson’s linear correlation (R), root mean squared error (RMSE), and mean absolute error (MAE) evaluations were conducted. Furthermore, the Bland–Altman plot was conducted for the further evaluation. R, RMSE, and MAE evaluations are shown in Equations (1)–(3).

(1) $$R_{x,y}=\frac{\sum_{i=1}^{n}\left(x_{i}-\bar{x}\right)\left(y_{i}-\bar{y}\right)}{\sqrt{\left[\sum_{i=1}^{n}{\left(x_{i}-\bar{x}\right)}^{2}\sum_{i=1}^{n}{\left(y_{i}-\bar{y}\right)}^{2}\right]}}$$

(2) MAE=1n∑i=1n|xi−yi|

(3) RMSE=1n∑i=1n(xi−yi)2

where $x_{i}$ is the reference, $y_{i}$ is the predicted result, n is the number of samples, $\bar{x}$ is the mean of the reference, and $\bar{y}$ is the mean of the predicted result.

## 3. Results

The multi-atrous deep convolutional autoencoder models were applied to both cardiovascular and cerebral hemodynamics systems using a single ECG signal. Pearson’s linear correlation, root mean squared error, and mean absolute error evaluations were deployed as performance indicators. Finally, a Bland-Altman plot was conducted to investigate the performance of the deep learning for both cardiovascular and cerebral hemodynamics models. The models initially generated the hemodynamics waveform. This waveform generating system is fundamental due to it accommodating the morphological cardiovascular conditions [44]. Finally, from the generated waveform, the systolic, diastolic, or the mean values were extracted.

### 3.1. Cardiovascular Hemodynamics

**Figure 3:**
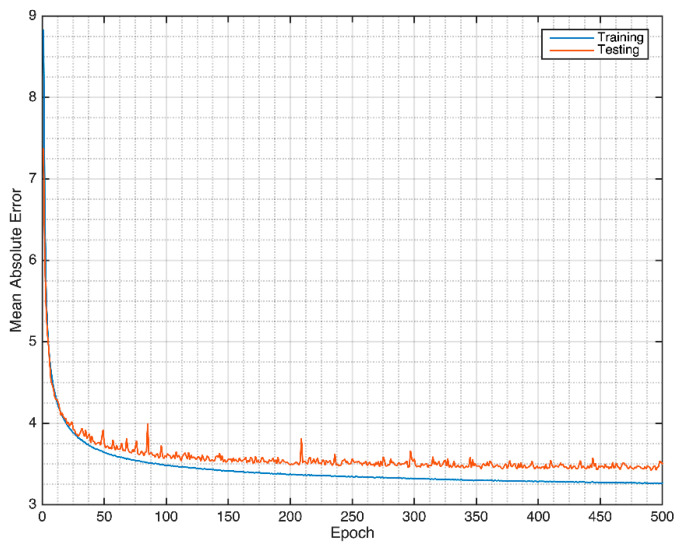
MA-UDCAE model convergence for cardiovascular hemodynamics.

The Massachusetts General Hospital/Marquette Foundation (MGH/MF) Waveform Database was used for this cardiovascular hemodynamics system. The ECG was used to generate the ABP, CVP, and PAP signals. The model was trained for 500 epochs, as shown in Figure 3. As it can be seen, the model starts to saturate at 400 epochs. The training and validation curves are relatively stable. The 5-fold cross-validation results show 0.942 ± 0.001, 7.833 ± 0.061 mmHg, and 4.959 ± 0.044 mmHg, respectively, for the R, RMSE, and MAE of ABP waveform evaluations. Furthermore, the CVP evaluation records are: 0.852 ± 0.002, 3.155 ± 0.018 mmHg, 2.024 ± 0.020 mmHg for R, RMSE, and MAE, respectively. Finally, for the PAP waveform evaluation, the evaluations of R, RMSE, and MAE are 0.873 ± 0.002, 4.853 ± 0.031 mmHg, and 3.263 ± 0.034 mmHg. The detail about the waveform evaluations is given in Table 1.

Furthermore, the systolic and diastolic pressures were calculated for the ABP and PAP. Meanwhile, the mean CVP value was also predicted from the waveform. For the ABP system, the systolic and diastolic pressures, the R evaluations are 0.894 ± 0.004 and 0.881 ± 0.005, respectively. The RMSE evaluations are 8.997 ± 0.401 mmHg and 4.726 ± 0.129 mmHg, respectively. The MSE evaluations are, respectively, 6.645 ± 0.353 mmHg and 3.210 ± 0.104 mmHg. Furthermore, for the PAP system, the systolic and diastolic pressures, the R evaluations are 0.864 ± 0.003 mmHg and 0.817 ± 0.006 mmHg, respectively. The RMSE evaluations are 5.833 ± 0.075 mmHg and 4.360 ± 0.179 mmHg, respectively. The MSE evaluations are 3.847 ± 0.136 mmHg and 2.964 ± 0.181 mmHg, respectively. Meanwhile, the mean CVP evaluations are 0.916 ± 0.001, 2.220 ± 0.039 mmHg, and 1.329 ± 0.036 mmHg for R, RMSE, and MSE, respectively. The details about these evaluations are shown in Table 2, Table 3 and Table 4. 

**Figure 4:**
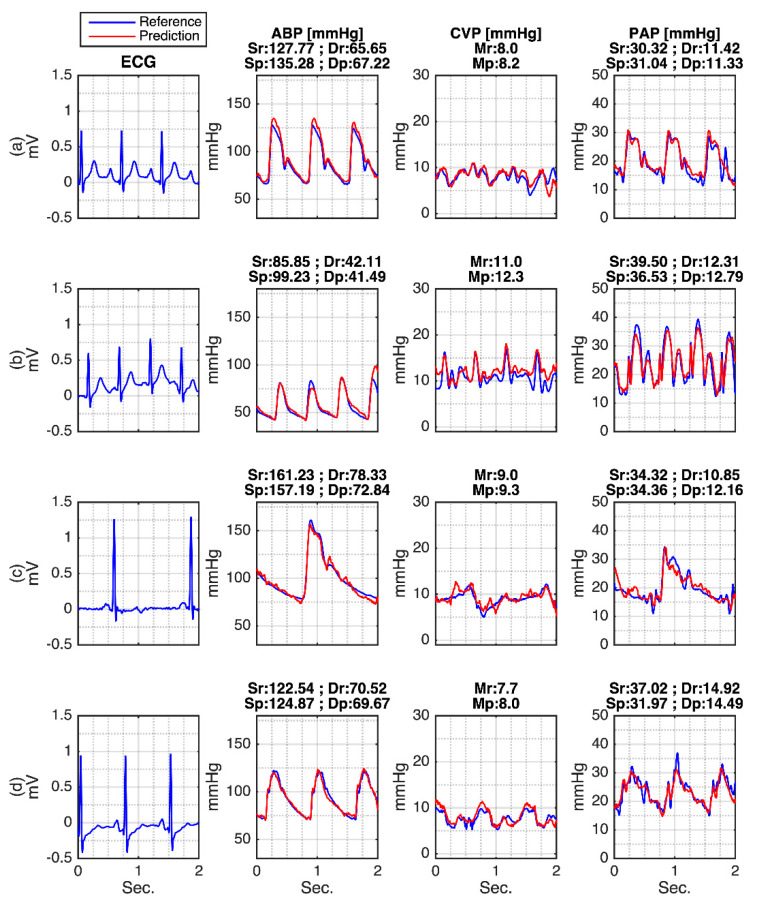
MA-UDCAE-generated waveforms of ABP, CVP, and PAP: (**a**) Normal ECG with normal hemodynamics; (**b**) Fast ECG with abnormal hemodynamics; (**c**) Slow ECG with abnormal hemodynamics; (**d**) Normal ECG with abnormal hemodynamics. Note: Systolic reference (Sr); diastolic reference (Dr); systolic prediction (Sp); diastolic prediction (Dp); mean reference (Mr); mean prediction (Mp).

The waveform evaluation result is shown in Figure 4. This figure also investigates the information of systolic, diastolic, and mean pressures from ABP, CVP, and PAP. Most importantly, the ability of MA-UDCAE deals with several conditions of the ECG, ABP, CVP, and PAP signals within two seconds and is also given in this figure. From Figure 4a, it can be seen how a relatively normal heartbeat generates normal ABP, CVP, and PAP signals. From this figure, the model predicts accurate systolic, diastolic, and mean values. In Figure 4b, ECG has a higher heart rate. In this case, the subject has very low ABP information, high CVP, and high PAP measures. A high error is given from the systolic ABP in the last period of the CVP waveform. However, the PAP is relatively good. Normal heart rate ECG is given in Figure 4c. However, this subject has relatively high systolic ABP, and relatively high CVP and PAP. Furthermore, Figure 4d shows relatively higher ECG heartbeat for generating normal ABP, normal CVP, and high PAP. It can be seen that the systolic PAP has a relatively high error.

**Figure 5:**
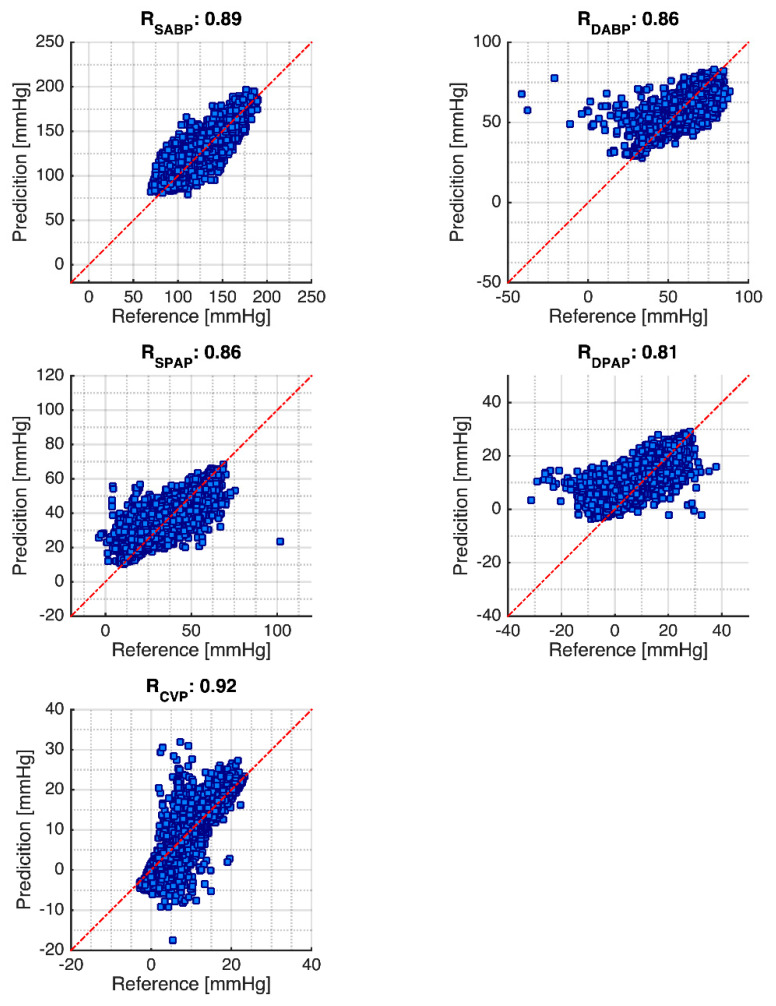
Pearson’s linear correlation coefficient results. Note: Red dotted line is the diagonal line.

**Figure 6:**
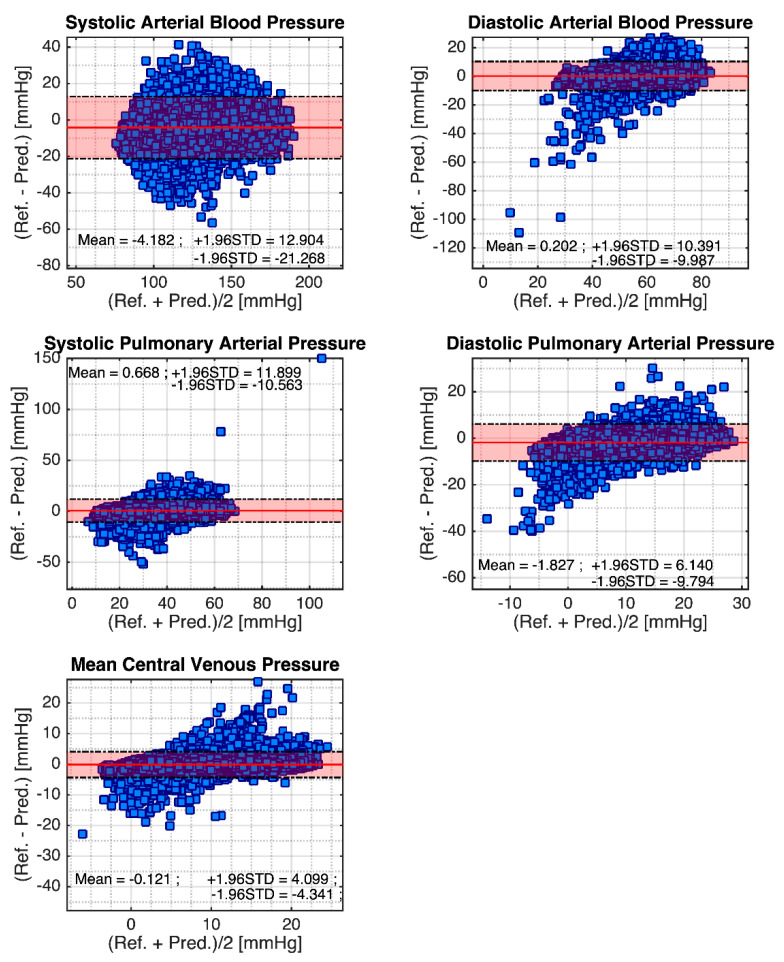
Bland–Altmann plots for cardiovascular hemodynamics system. Note: Red line is the mean, and dash-dot lines are ±1.96 standard deviations.

Finally, the Pearson’s linear correlation and Bland–Altman plot are given in Figure 5 and Figure 6. Figure 5 shows the Pearson’s linear correlation result. It can be seen that the correlation coefficient of systolic ABP and diastolic ABP are 0.89 and 0.86, respectively. Meanwhile, the SPAP and DPAP are, respectively, 0.86 and 0.81. Finally, the mean CVP is 0.92. Figure 6 shows the Bland–Altman plot for the cardiovascular hemodynamics system. For systolic ABP, the reference and prediction have the mean difference of −4.182 mmHg, −1.96 STD of −21.268 mmHg, and +1.96 STD of 12.904. For the diastolic ABP, it has a mean difference of 0.202 mmHg, −1.96 STD of −9.987 mmHg, and +1.96 STD of 10.391. Furthermore, for the systolic PAP, the mean difference is 0.668 mmHg, −1.96 STD of −10.563 mmHg, and +1.96 STD of 11.899 mmHg. For diastolic PAP, the mean difference is −1.827 mmHg, −1.96 STD of 6.140 mmHg, and +1.96 STD of −9.794 mmHg. Finally, the mean CVP has mean difference of −0.121 mmHg, −1.96 STD of 4.099, and +1.96 STD of −4.341 mmHg. The graphs show some negative values appearing in the prediction. This situation likely happens due to the complexity of patient monitoring, and the method for selecting the systolic and diastolic peaks using maximum and minimum values. 

### 3.2. Intracranial Pressure

**Figure 7:**
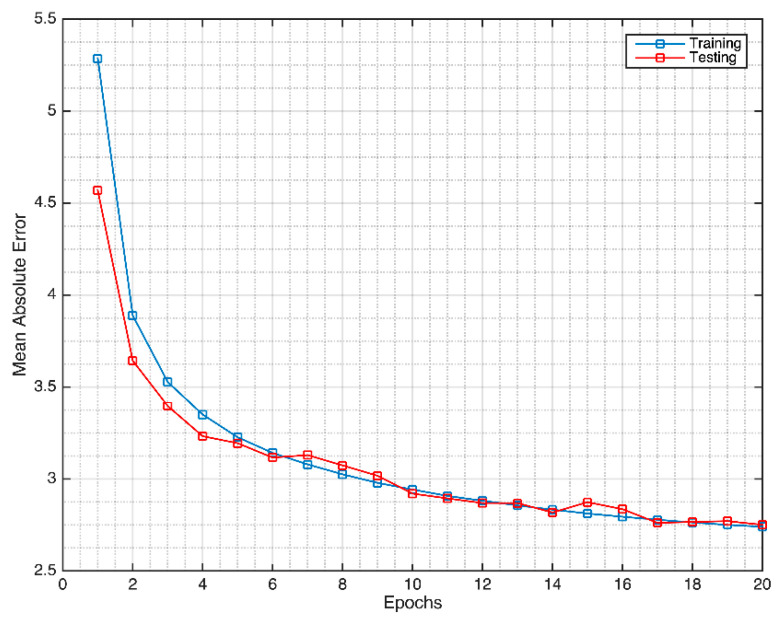
The MA-UDCAE model convergence for intracranial pressure.

For the intracranial pressure evaluation, the Cerebral Hemodynamic Autoregulatory Information System Database (CHARIS DB) was utilized. The MA-UDCAE model was also applied for the ECG signal to understand the ICP pattern. For the evaluation of the deep learning system from the ECG to ICP, the MA-UDCAE model was applied. This system was conducted for 20 epochs. The mean absolute error was used for the evaluation. The ICP training convergence is shown in Figure 7.

**Figure 8:**
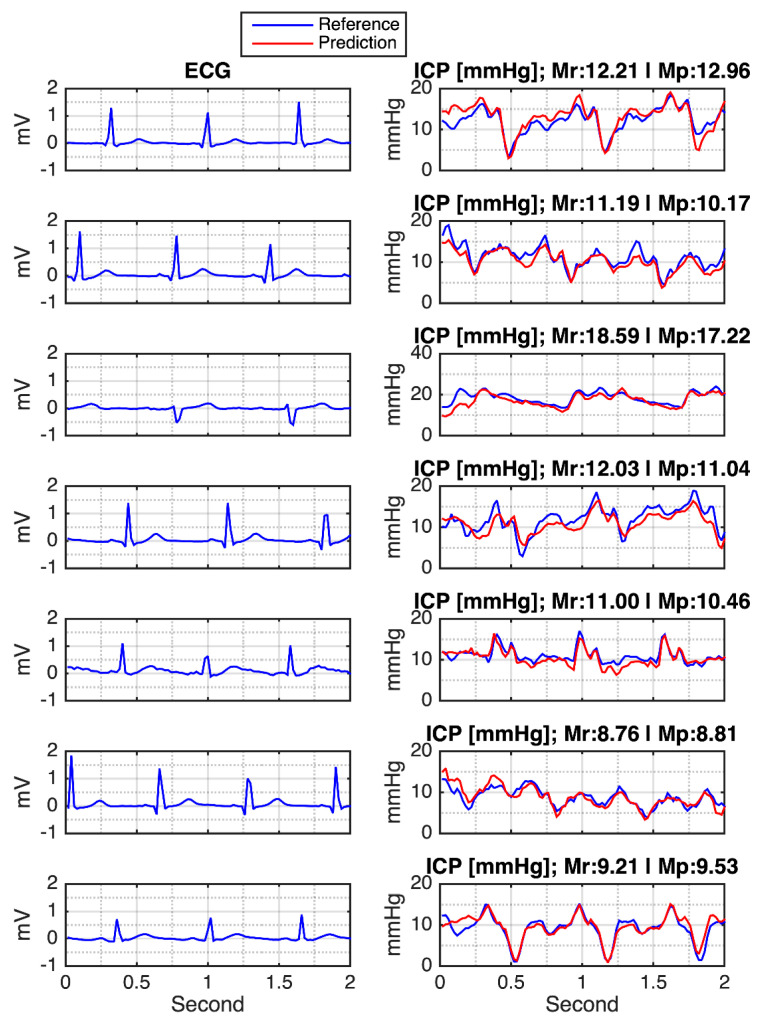
MA-UDCAE-generated waveform of ICP. Note: Mean reference (Mr); mean prediction (Mp).

After the training season, the testing data were subsequently evaluated into the trained model. Some of the ECG-generated ICP waveforms can be seen in Figure 8. These results are selected based on the variation of the reference ICP. From this figure, it can be seen that the model is not only reasonably robust in handling the data that has either a relatively low or high ICP index, but also gives some information on how the model decodes the ICP waveform. 

**Figure 9:**
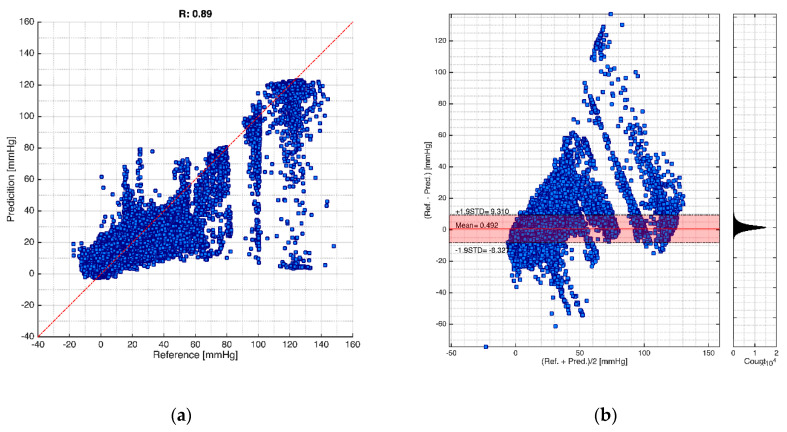
Intracranial pressure evaluations: (**a**) Pearson’s linear correlation result for intracranial pressure evaluation. Note: Red dotted line is the diagonal line; (**b**) Bland–Altmann plot for intracranial pressure evaluation. Note: Red line is the mean, and dash-dot lines are for ±1.96 standard deviations.

Furthermore, in more detail, for the intracranial pressure waveform evaluations, the cross-validation results generate 0.887 ± 0.003, 5.306 ± 0.041 mmHg, and 2.765 ± 0.026 mmHg, respectively, for R, RMSE, and MAE. For the mean ICP evaluation, the R, RMSE and MAE evaluations are 0.914 ± 0.003, 4.582 ± 0.044 mmHg, and 2.404 ± 0.043 mmHg, as shown in Table 5. The intracranial evaluations for Pearson’s linear correlation and Bland–Altman are shown in Figure 9. Pearson’s linear correlation for the ICP is 0.89, as given in Figure 9a. From this figure, it can be seen that the model likely starts to generate a bigger error when dealing with an ICP higher than 30 mmHg. In addition, Figure 9b shows the Bland–Altman plot for ICP evaluations. Results in Figure 9b support Pearson’s linear correlation. As it can be seen, the mean difference is 0.492 mmHg, −1.96 STD of −8.32 mmHg, and +1.96 STD of 9.310 mmHg. Even though it has relatively low prediction error, the model gives lower accuracy on higher ICP measures. The entire ICP evaluation is shown in Table 5, indicating that all evaluations of R, MAE, and RMSE have fairly low standard deviation values. However, some negative values can be seen appearing in the prediction. This situation likely happens due to the complexity of the patient data. 

## 4. Discussion

The novelty in this study is the utilization of a non-invasive ECG signal to investigate cardiovascular and cerebral hemodynamics. Initially, the models generated the cardiovascular hemodynamics ABP, PAP, and CVP from MGHDB. This study used two databases from PhysioNet: Massachusetts General Hospital/Marquette Foundation (MGH/MF) Waveform Database and Cerebral Hemodynamic Autoregulatory Information System Database (CHARIS DB). The MA-UDCAE deep learning model was deployed for modeling these systems. 

Most of the previously conducted studies as shown in Table 6 utilized PPG signal as an input of the model. Slapničar et al. [45] used PPG signal of 510 subjects of MIMIC III from a PhysioNet and ResNet-based model to investigate hypertension. This previous study had a MAE of 9.43 mmHg and 6.88 mmHg, respectively, for SBP and DBP. Chowdhury et al. administered the PPG signal of 126 subjects and a regression model. Their results achieved a correlation coefficient of 0.95 and 0.96, respectively, for SBP and DBP, and MAE of SBP and DBP are, respectively, 3.02 mmHg and 1.74 mmHg [46]. Meanwhile, Zadi et al. [47] applied the ARMA algorithm to PPG signals from 15 subjects. They achieved an RMSE of 7.21 mmHg and 5.12 mmHg for SBP and DBP, respectively. However, none of these studies provides information about the continuous waveform evaluation.

Several previous studies, shown in Table 6, have utilized PPG signal as their input, and investigated the waveform evaluation. Sideris et al. [9] performed evaluation of continuous blood pressure using PPG signals with the LSTM method, delivering an RMSE of 6.04 ± 3.26 mmHg, 2.58 ± 1.23 mmHg, and 1.98 ± 1.06 mmHg, respectively, on SBP and DBP evaluations, and a waveform correlation coefficient of 0.95 ± 0.045. Meanwhile, Sadrawi et al. [10] utilized PPG for arterial blood pressure estimation, reporting 0.984 linear correlation and MAEs of 2.54 mmHg and 1.48 mmHg for the systolic and diastolic, respectively. Furthermore, Aguirre et al. [48] utilized PPG signal of 1131 subjects from the PhysioNet database using the Seq2seq algorithm to evaluate the hemodynamics. This study also investigated the arterial blood pressure waveform. The generated waveform had a correlation coefficient of 0.98. Meanwhile, the MAE of systolic and diastolic were 12.08 mmHg and 5.56 mmHg, respectively [48].

On the other hand, prior studies as shown in Table 6 also correlated ECG signal with blood pressure estimation. Tanveer et al. [6] used a combination of ECG and PPG, utilizing ANN and LSTM. This study reported 0.93 mmHg and 0.52 mmHg for SBP and DBP, respectively, on the MAE estimations. This earlier study has very high correlation coefficients, 0.999 and 0.998, respectively, for the SBP and DBP. Another study by Wu et al. [7] also used ECG and PPG with the hybrid ANN method, and produced 3.404 mmHg and 3.289 mmHg, respectively, for SBP and DBP. Meanwhile, a study by Eom et al. [8] conducted an investigation of multiple input signals, ECG, PPG, and BCG, combined with CNN, Bi-GRU, and attention methods. This previous study produced an MAE of 4.06 ± 4.04 and 3.33 ± 3.42, respectively, for SBP and DBP evaluations. Meanwhile, it had 0.52 and 0.49 for the R^2^ evaluations for the SBP and DBP, respectively. The study that only used the ECG signal to investigate hypertension was conducted by Fan et al. [23]. This previously conducted study reported the MAE for the systolic and diastolic as 7.69 and 4.36 mmHg, respectively.

For a comparison of intracranial pressure evaluation, this study is compared to several sub-studies, given in Table 7. These previously conducted works were undertaken by utilizing the ABP and cerebral blood flow velocity (CBFV). Imaduddin et al. [3] used these signals to estimate the ICP from 13 patients with the Bayesian system. This previous study provided an RMSE of 3.7 mmHg. Another study conducted by Jaishankar et al. [4] had RMSEs of 5.1 mmHg and 4.5 mmHg, respectively, for 13 pediatric and 5 adult subjects. This prior study utilized the spectral method. Even though the CBFV is a non-invasive system, the ABP is classified as an invasive technique. This study reported 4.582 ± 0.044 mmHg RMSE from five cross-validation systems. This result is slightly inferior compared to the previously compared study. However, the novelty of this study is the utilization of the non-invasive ECG signal as the input signal to generate the ICP waveform with further evaluation of the mean ICP.

The association between ECG and hemodynamics is related to the fact that ECG and ABP have a very close relationship in time-related terms, especially in the evaluation of the heart-related system. This may have some similarity in CVP, PAP, and ICP due to the blood circulation. Furthermore, the systolic peak and the R peak intervals are shifted in some transmitting time. However, more investigation is required to be performed morphologically for the full cycle between the ECG and cardiovascular and cerebral hemodynamics signals, especially for CVP and ICP.

In order to evaluate the network performance, an ablation study was performed. The autoencoder structure was utilized based on [10]. From this previous study, specifically for hemodynamics, the UNET-based model provided a better result compared to the classical autoencoder network. Hence, based on reference [10], the structure was modified by utilizing the multi-atrous system. For simplification, this study investigates the results only at 25 and 5 epochs, respectively, for the cardiovascular and cerebral hemodynamics systems.

**Figure 10:**
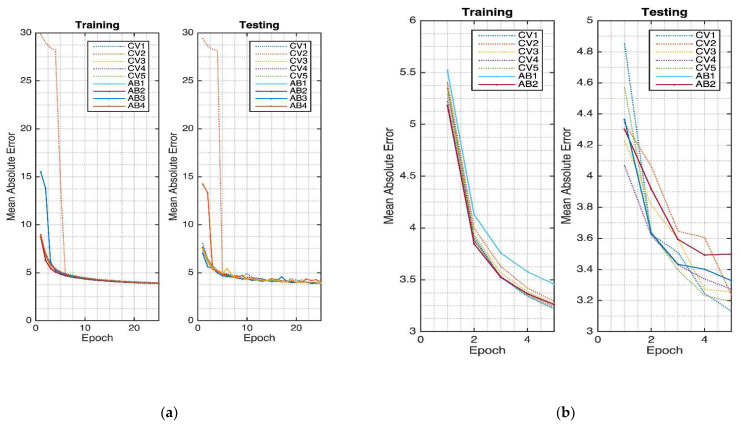
Ablation network: (**a**) cardiovascular hemodynamics; (**b**) cerebral hemodynamics.

The ablation network evaluation was also conducted in our study, given in Figure 10. For the cardiovascular system, the ablated network convergence is shown in Figure 10a. Several ablations were conducted. The decreased layers are sequentially the layer connections between DP_04 and CV_05, DP_03 and CV_06, DP_02 and CV_07, DP_01 and CV_08, and the input layer and CV_09. From this figure, it can be seen that the first ablated layer attaching between DP_03 and CV_06 tends to have a faster convergence rate compared to others. However, the results are relatively similar at the 25th epoch. For testing, the deleted connection systems tend to have some oscillation during this early period compared to the non-ablated networks.

The cerebral hemodynamics ablation study was conducted by removing the concatenating layers. The first deleted layer is the connection between ME_02 and CV_04. The next one is between ME_01 and CV_05. The results are shown in Figure 10b, where in the training phase, most of the cross-validation models without ablation have relatively lower MAE values at the 5th epoch compared to the ablated models. In this system, the deleted connection models tend to saturate earlier compared to the system without any ablated connection. Furthermore, the ablated networks also have a slower convergence rate at the testing.

This study has several limitations due to the selection of systolic and diastolic techniques being sensitive to noise. In more detail, the noise has a positive effect on the systolic and a negative effect on the diastolic pressure. This phenomenon will generate higher error during the evaluation.

Due to the data limitations, one of the main limitations of this study is the testing data separated randomly from all patients instead of patient-based partition. Even though the data were not fully interpreted, this strategy will let the model learn and memorize some patterns of the output through the input. Another limitation is the high dependency on the quality of the ECG signal. The mitigations of the dataset unbalancing can be investigated further [49] and other deep learning structures can be applied in future [50,51,52].

**Figure 11:**
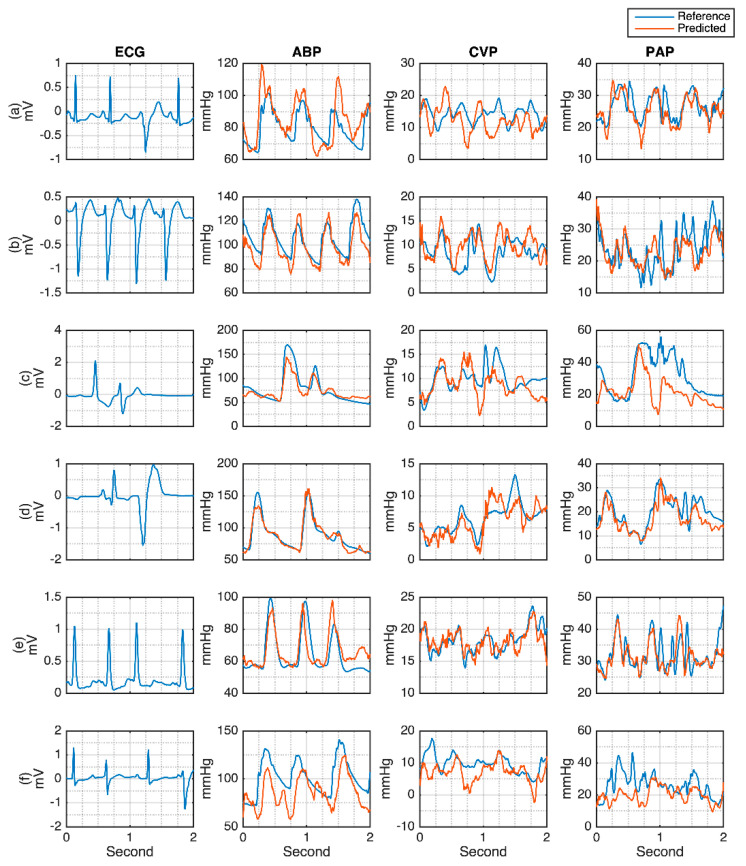
Arrhythmic ECG predictions for cardiovascular hemodynamics. (**a**) rapid R waves with premature beat; (**b**) rapid heart rate with downward R waves; (**c**) Slow heart rate with relatively small downward R wave; (**d**) Slow heart rate with bigger downward R wave; (**e**) Rapid upward R wave with irregular interval; (**f**) Rapid heart rate with multiple downward R wave.

**Figure 12:**
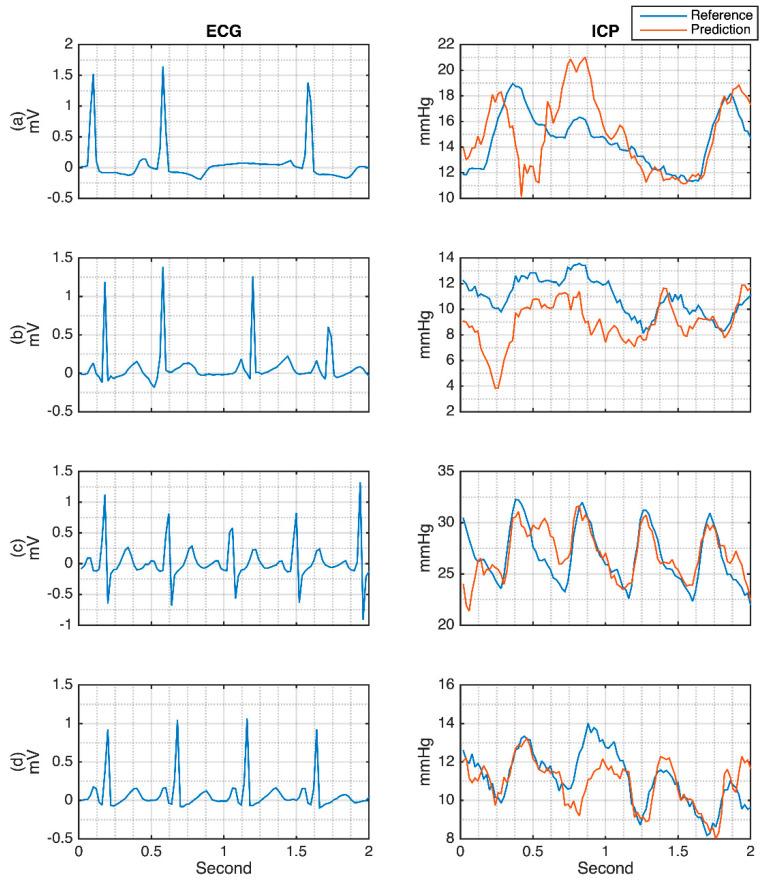
Arrhythmic ECG evaluations for the intracranial pressure. (**a**) Irregular interval heart rate; (**b**) Irregular and rapid heart rate; (**c**) Rapid heart rate with downward shifted R waves; (**d**) Rapid heart rate.

There are also concerns regarding the evaluations of arrhythmia conditions. In this study, some of the arrhythmia ECG factors affecting the cardiovascular and cerebral hemodynamics systems have been investigated. In addition, the cardiovascular hemodynamics dataset was well annotated for some arrhythmia conditions. However, for the cerebral hemodynamics, the heartbeat in the dataset was not labeled. Nevertheless, it is still possible for the rapid and irregular beats to be investigated. The premature ventricular contraction (PVC) and supraventricular premature/ectopic beats were evaluated. Figure 11 shows how arrhythmia affects the cardiovascular hemodynamics. It can be seen that most of the abnormal ECG signals are relatively good in generating the ABP. However, there are some shifts in the SBP and DBP, as shown in Figure 11a,f. Bigger differences are shown for CVP and PAP signals. The generated ICP from the abnormal ECG is shown in Figure 12. From Figure 12a,b, the R-R interval irregularity has a worse effect on the ICP prediction compared to the arrhythmia generated from the rapid R interval. However, deeper evaluation with many more additional arrhythmia cases to investigate the effect of arrhythmia on the hemodynamics should be performed in the future.

Since this study is preliminary and a proof of concept, it may not provide superior results compared to previous studies that used multi-input signals such as the combination of PPG and ECG, in which the shape of the PPG signal is much more identical to ABP signal compared to the ECG and ABP signal. However, as with preliminary and proof of concept studies, this study can be a finding of using the ECG in hemodynamics investigations. Finally, the cross-validation test using the same group of subjects in our current study was inner loop cross-validation, which only rotates the validation data and training data. However, to make the results more convincing, the testing data need to be rotated into training and validation, which is known as outer loop cross-validation, and is to be considered in future works. In addition, the pre-processing of the data was initially filtered manually by visualization. This manual filter may not be practical for the study. We still need further investigation about using automatic filtering to filter all these vital signs for future work.

## 5. Conclusions

In order to design the most precise health monitoring system to solve the hemodynamics system in ICU, in this study, as a proof of concept, a deep convolutional autoencoder system has been implemented for a non-invasive system by only using a single ECG signal and utilizing two databases for cardiovascular and cerebrovascular hemodynamics systems. For the preliminary result, it can be seen that ECG has great potential in generating the ABP, CVP, PAP, and ICP waveform, as well as their essential information for the extensive evaluations. 

## Figures and Tables

**Table 1:** Waveform evaluations of cardiovascular hemodynamics.

CV	ABP			CVP			PAP		
	R	RMSE (mmHg)	MAE (mmHg)	R	RMSE (mmHg)	MAE (mmHg)	R	RMSE (mmHg)	MAE (mmHg)
1	0.941	7.874	4.950	0.850	3.168	2.032	0.871	4.863	3.270
2	0.942	7.915	5.036	0.854	3.124	1.994	0.874	4.805	3.207
3	0.941	7.819	4.927	0.851	3.156	2.015	0.872	4.863	3.262
4	0.942	7.797	4.938	0.852	3.165	2.045	0.872	4.888	3.295
5	0.943	7.761	4.945	0.855	3.162	2.032	0.875	4.847	3.281
Mean	0.942	7.833	4.959	0.852	3.155	2.024	0.873	4.853	3.263
STD	0.001	0.061	0.044	0.002	0.018	0.020	0.002	0.031	0.034

**Table 2:** Systolic and diastolic arterial blood pressure evaluations.

CV	Arterial Blood Pressure					
	R		RMSE (mmHg)		MAE (mmHg)	
	SBP	DBP	SBP	DBP	SBP	DBP
1	0.890	0.875	8.980	4.906	6.517	3.344
2	0.894	0.884	9.640	4.575	7.260	3.093
3	0.892	0.878	8.926	4.782	6.593	3.217
4	0.895	0.881	8.905	4.638	6.482	3.123
5	0.900	0.888	8.534	4.730	6.371	3.272
Mean	0.894	0.881	8.997	4.726	6.645	3.210
STD	0.004	0.005	0.401	0.129	0.353	0.104

**Table 3:** Mean central venous pressures.

CV	Central Venous Pressure		
	R	RMSE (mmHg)	MAE (mmHg)
1	0.916	2.232	1.332
2	0.918	2.156	1.277
3	0.915	2.219	1.312
4	0.916	2.228	1.369
5	0.917	2.264	1.353
Mean	0.916	2.220	1.329
STD	0.001	0.039	0.036

**Table 4:** Systolic and diastolic pulmonary arterial pressure evaluations.

CV	Pulmonary Arterial Pressure					
	R		RMSE (mmHg)		MAE (mmHg)	
	SBP	DBP	SBP	DBP	SBP	DBP
1	0.859	0.818	5.864	4.295	3.863	2.892
2	0.864	0.813	5.769	4.457	3.766	3.042
3	0.863	0.810	5.819	4.329	3.817	2.938
4	0.864	0.817	5.766	4.598	3.717	3.218
5	0.868	0.827	5.947	4.122	4.070	2.730
Mean	0.864	0.817	5.833	4.360	3.847	2.964
STD	0.003	0.006	0.075	0.179	0.136	0.181

**Table 5:** Intracranial pressure evaluations.

CV	R		RMSE (mmHg)		MAE (mmHg)	
	Waveform	Mean	Waveform	Mean	Waveform	Mean
1	0.890	0.917	5.330	4.592	2.786	2.435
2	0.884	0.912	5.342	4.603	2.762	2.398
3	0.885	0.910	5.333	4.637	2.758	2.393
4	0.888	0.914	5.254	4.550	2.792	2.453
5	0.889	0.915	5.269	4.526	2.726	2.343
Mean	0.887	0.914	5.306	4.582	2.765	2.404
STD	0.003	0.003	0.041	0.044	0.026	0.043

**Table 6:** Cardiovascular hemodynamics evaluations. Note: RMSE and MAE are in mmHg.

Studies	Dataset	Input Signal	Cont. ABP	Method	Perf. Eval.	Waveform	SBP	DBP
Tanveer et al. [6]	39 subjects, MIMIC, PhysioNet	ECG + PPG	No	ANN + LSTM	RMSE	N/A	1.26	0.73
					MAE	N/A	0.93	0.52
					R	N/A	0.999	0.998
Wu et al. [7]	27 subjects	ECG + PPG	No		RMSE	N/A	3.404	3.289
Eom et al. [8]	15 subjects	ECG + PPG + BCG	No	CNN + Bi-GRU + Attention	MAE	N/A	4.06 ± 4.04	3.33 ± 3.42
					R^2^	N/A	0.52	0.49
Sideris et al. [9]	42 subjects, MIMIC, PhysioNet	PPG	Yes	LSTM	RMSE	6.04 ± 3.26	2.58 ± 1.23	1.98 ± 1.06
					R	0.95 ± 0.05	N/A	N/A
Sadrawi et al. [10]	18 Patients, NTUH, Taiwan	PPG	Yes	GDCAE	RMSE	3.46	3.41	2.14
					MAE	2.33	2.54	1.48
					R	0.984	0.981	0.979
Fan et al. [30]	MIMIC II, PhysioNet	ECG	No	BiLSTM + FCN	RMSE	N/A	12.3	6.88
					MAE	N/A	7.69	4.36
Slapničar et al. [45]	510 subjects, MIMIC III, PhysioNet	PPG	No	Spectro temporal ResNet	MAE	N/A	9.43	6.88
Chowdhury et al. [46]	222 records, 126 subjects	PPG	No	Gaussian process regression	RMSE	N/A	6.74	3.59
					MAE	N/A	3.02	1.74
					R	N/A	0.95	0.96
					MSE	N/A	45.49	12.89
Aguirre et al. [48]	1131 subjects, MIMIC, PhysioNet	PPG	Yes	Seq2seq + Attention	RMSE	8.67	15.96	7.4
					MAE	7.39	12.08	5.56
					R	0.98	N/A	N/A
					R^2^	N/A	0.39	0.41
Zadi et al. [47]	15 subjects	PPG	No	ARMA	RMSE	N/A	7.21	5.12
Proposed	250 subjects, MGH/MF, PhysioNet	ECG	Yes	MA-UDCAE	RMSE	7.83 ± 0.06	8.99 ± 0.40	4.73 ± 0.13
					MAE	4.95 ± 0.04	6.64 ± 0.35	3.21 ± 0.10
					R	0.94 ± 0.00	0.89 ± 0.00	0.88 ± 0.01

**Table 7:** Intracranial pressure evaluations. Note: RMSE and MAE are in mmHg.

Studies	Dataset	Input Signal	Method	Performance Evaluation	Mean ICP (mmHg)
Imaduddin et al. [3]	13 subjects	ABP + CBFV	Bayesian model	RMSE	3.7
Jaishankar et al. [4]	13 pediatric subjects	ABP + CBFV	Spectral approach	RMSE	5.1
	5 adult subjects				4.5
Proposed	13 subjects, CHARISD, PhysioNet	ECG	MAUDCAE	RMSE	4.582 ± 0.044
				MAE	2.404 ± 0.043
				R	0.914 ± 0.003

## Data Availability

This study utilizes the publicly available dataset, from https://physionet.org, accessed on 14 January 2021.
